# Hand-foot syndrome associated with use of sorafenib in a patient with papillary thyroid cancer: a case report

**DOI:** 10.1186/1472-6823-14-26

**Published:** 2014-03-19

**Authors:** Carlos Velandia-Carrillo, Edwin Wandurraga-Sánchez, Diego Gómez-Abreo

**Affiliations:** 1 Universidad Autónoma de Bucaramanga-UNAB, Tower B, Eighth Floor, Office 806 Street 155 A 23-09, Urbanización El Bosque, Floridablanca, Santander, Colombia; 2 Department of Endocrinology, Fundación Oftalmológica de Santander-FOSCAL Clinic, Universidad Autónoma de Bucaramanga-UNAB, Bucaramanga, Colombia; 3 Tower B, Eighth Floor, Office 806 Street 155 A 23-09, El Bosque, Floridablanca, Santander, Colombia; 4 Department of Clinical Oncology, Cal ONCOLOGICOS, Bucaramanga, Colombia; 5 González Valencia Street # 55b-07, Bucaramanga, Santander, Colombia

**Keywords:** Hand-foot syndrome, Palmo-plantar Erythrodysesthesia, Sorafenib, Thyroid Cancer, Tyrosin kinase inhibitor, Targeted therapies

## Abstract

**Background:**

Hand-foot syndrome (HFS), also known as palmar-plantar Erythrodysesthesia (PPE), acral erythema or Burgdorf reaction, is a dermatologic toxic reaction to certain chemotherapies, including sorafenib. A high incidence of adverse events is already described in dermatological clinical trials of this drug, but its use in medical practice, common in the patient with metastatic thyroid carcinoma has not yet been reported. Sorafenib (BAY 43-9006) is an orally administered multi-level kinase inhibitor, approved for treatment of solid tumors such as renal cell carcinoma, hepatocellular carcinoma and recently for metastatic thyroid carcinoma.

**Case presentation:**

We report a case of a 29 year old Latin woman diagnosed with papillary thyroid carcinoma, who was initially given a total thyroidectomy, central and bilateral neck lymph node removal followed by a radioiodine therapy. Subsequent evaluation indicated locoregional progressive disease and metastatic involvement in both lungs. Following this, the patient was prescribed 200 mg of sorafenib administered every 12 hours, but after four days, she presented with a skin reaction compatible with hand-foot syndrome. After discontinuation of the therapy, this reaction ceased.

**Conclusion:**

Sorafenib as a new therapeutic option for patients with radioactive iodine (RAI)-resistant metastatic differentiated thyroid cancer, it is important that clinicians are fully aware of the potential adverse effects. All patients on Sorafenib therapy should be educated to recognize the first symptoms to obtaining the maximal benefit from this anti-neoplastic rescue therapy.

## Background

Thyroid cancer is the most common endocrine neoplasia globally, with approximately 8.7 cases per 100,000 populations [1]. Although it usually has a favorable prognosis, an incidence of 0.5 deaths per 100,000 populations is estimated [2]. Surgery is considered the first-line therapy, while radioactive iodine has played an important role over the years in postsurgical remnant ablation and therapy of metastatic distant disease [3,4]. However, the therapeutic options for patients with advanced radioactive iodine (RAI)-refractory desease are limited. Doxorubicin had been accepted by most countries as the only systemic treatment, which has traditionally been a limited option due to the rarity of complete and partial responses and because of its significant toxicity [5]. Recently the US department of food and drug administration (FDA) approved the use of Sorafenib (BAY43 -9006) for the treatment of differentiated thyroid cancer metastases unresponsive to radioiodine treatment [6]. This drug had previously been approved for the treatment of hepatocellular carcinoma [7] and advanced renal carcinoma [8].

Sorafenib is a molecule capable of multi - level kinase inhibition, with particular activity against VEGFR and RAF (the RAF/MEK/ERK pathway), a major pathway involved in the proliferation of tumor cells [9], opposing c -RAF, B -Raf, c- KIT, FLT3, Platelet-derived growth factor receptor (PDGFR) α and β, vascular endothelial growth factor receptor (VEGFR) 1, 2, and 3, and various other kinases [10], giving this medicine both pro - apoptotic properties and anti-angiogenic effects. This is of special interest in the treatment of thyroid carcinomas [9], based on the understanding that mutations in the protein kinase BRAF, play a key role in carcinogenesis in up to 45% of cases of papillary thyroid cancer (PTC). Ras mutations and rearrangements in the RET (RET/PTC) gene are also involved in 10% and 5–30% of cases, respectively [11]. Adverse events with the use of Sorafenib for treatment of renal carcinoma [12] occur in up to 73% of patients as case of fatigue, rash/desquamation (up to 66% of patients), hand-foot skin reaction (up to 62% of patients) and diarrhea (up to 58% of patients). Similar prevalence is observed in treatment of hepatocellular carcinoma [13].

The safety and efficacy of Sorafenib in treating thyroid cancer were established in the Phase III DECISION study (Sorafenib in Locally Advanced or Metastatic RAI- Refractory Patients with Thyroid Cancer) in patients with locally recurrent or progressive metastatic differentiated thyroid cancer who had not responded to treatment with radioactive iodine [14]. This study randomized 417 patients to 400 mg of orally administered sorafenib taken every 12 hours, versus placebo. A partial response was observed in 12.2% of the patients receiving Sorafenib compared to 0.5% in the placebo group. Those receiving sorafenib also had a 5 month greater disease-free survival rate compared to the placebo group (10.8 vs. 5.8 months, HR 0.587, 95% CI [0.454, 0.758], p < 0.0001). 18% of patients discontinued medication due to adverse events in the Sorafenib group. Hand-foot syndrome which presented in 76.3% of patients and it was severe (grade 3) in 20.3%. Other symptoms reported were diarrhea in 68.6%, alopecia in 67.1%, rash/desquamation in 50.2%, fatigue in 49.8%, weight loss in 46.9% and hypertension in 40.6% [15]. In the present report we describe the case of a patient presenting with HFS after four days of 400 mg/day of oral Sorafenib. HFS toxicity is measured and recorded according to The National Cancer Institute (NCI) grading systems for dermatologic toxicities (NCI Common Terminology Criteria [CTC] v2.0 and CTC for Adverse Events [CTCAE] v3.0 (see Table 1[16]). However, these side effects required permanent discontinuation of the treatment in only 18.8% of patients [14,15,17].

**Table 1:** Severity of hand-foot syndrome

**Grade**	**Hand-foot syndrome**
1	Minimal skin changes or dermatitis (e.g., erythema, edema, or hyperkeratosis) without pain.
2	Skin changes (e.g., peeling, blisters, bleeding, edema, or hyperkeratosis) with pain; limiting instrumental activities of daily living.
3	Severe skin changes (e.g., peeling, blisters, bleeding, edema, or hyperkeratosis) with pain; limiting self care activities of daily living.

Consensus panel recommendations for different degrees of toxicity management follow:

For grade 1 HFS the use of moisturizing creams is recommended and patients should avoid hot water. Cotton gloves and socks can be worn at night to prevent further injury and to help retain moisture, no dose modifications of the Sorafenib are recommended in addition to continuation of prophylactic measures and a follow-up in the clinic. Keratolytics, such as urea 20%–40%, or salicylic acid 6% may be indicated.

For grade 2 treatment should continue as for grade 1 toxicity with the following additions. Consider applying Clobetasol 0.05% to erythematous areas twice daily, using topical analgesics such as Lidocaine 2% and assess kidney function before prescribing any systemic pain medications (e.g., nonsteroidal anti-inflammatory drugs, codeine, pregabalin). If necessary, consider a dose reduction to 50% of the full dose for a minimum of 7 days, up to 28 days, until the HFS reaches grade 1.

For grade 3 the recommendation is provide treatment as indicated for grades 1 and 2 and to include the following: Discontinue the Sorafenib for a minimum of 7 days until the HFS reaches grade 1 or 0, and then resume treatment at 50% of the full dose [18].

## Case presentation

**Figure 1:**
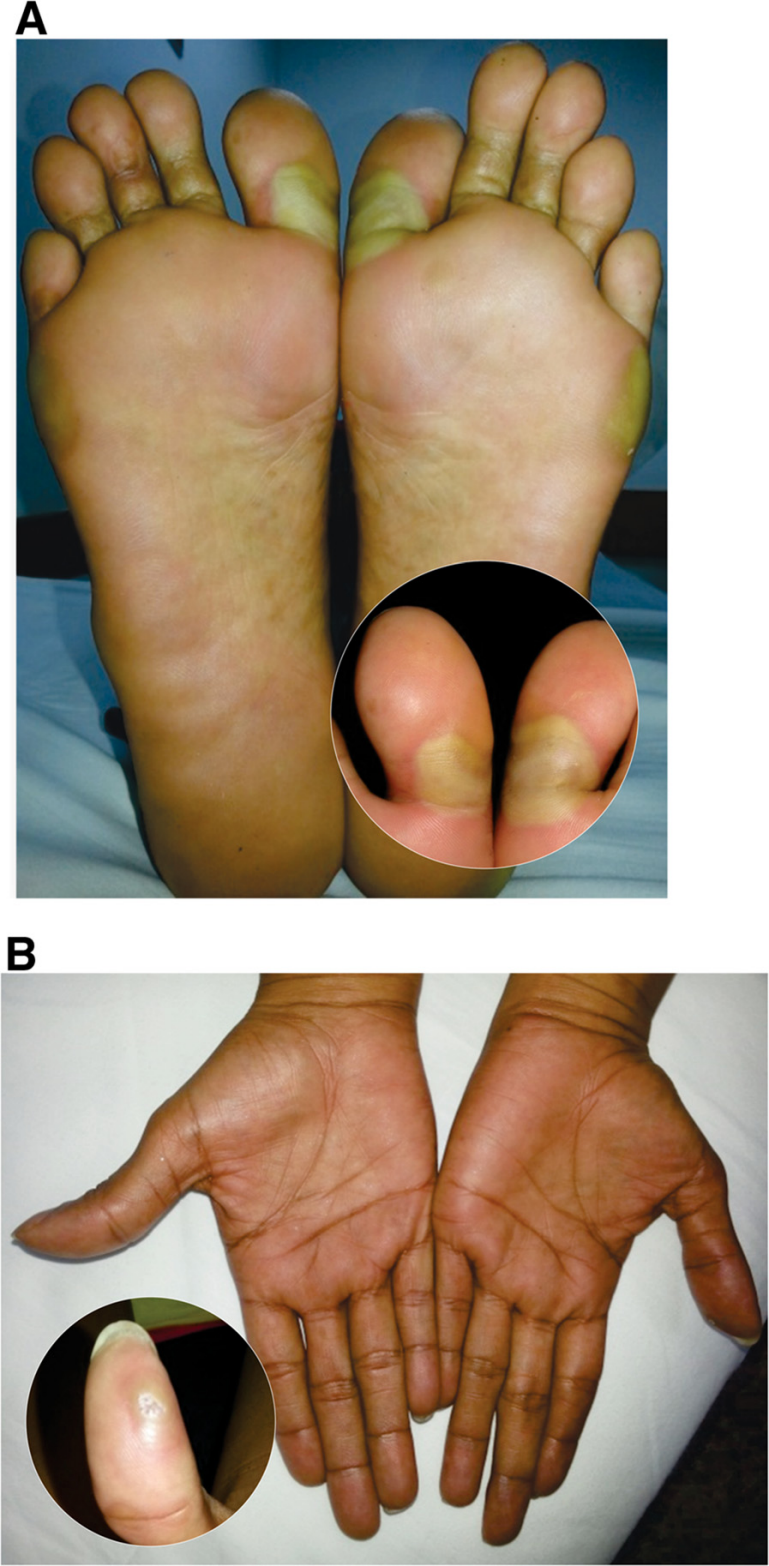
Hand-foot syndrome on the soles (A) and palms (B) (4 days after onset of sorafenib).

**Figure 2:**
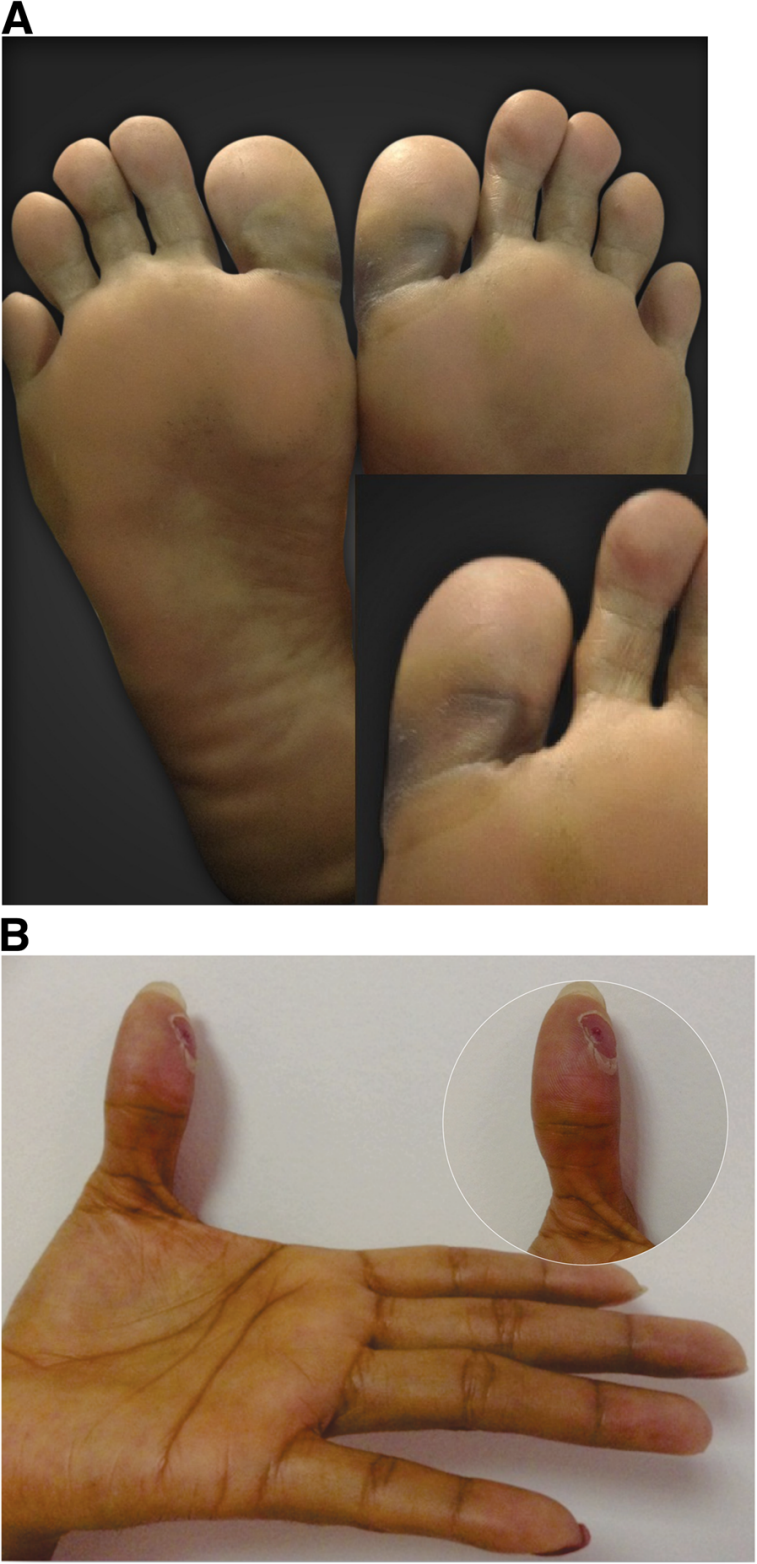
Hand-foot syndrome on the soles (A) and palms (B) (7 days after suspension of sorafenib and start symptomatic treatment).

A 29 year old Latina female with a history of papillary thyroid carcinoma was initially treated with a total thyroidectomy, central and bilateral neck lymph node removal and subsequently with radioiodine therapy. Following this, evaluation indicated locoregional progressive disease and micrometastatic involvement in both lungs. Therapy was then initiated with 200 mg of oral sorafenib every 12 hours. However, after 4 days the patient presented with cutaneous lesions in the sole of the foot and in the first finger of the left hand which was associated with a burning pain in soles and palms and an intolerance to contact with hot surfaces. She also had significant ambulatory limitations and patchy lesions were evident in several areas of the foot and yellow blisters on the lateral aspect of the foot, metatarsal region and on the proximal phalanx of the first finger of the left hand (Figure 1). Given these clinical findings the dermatological reaction was classified as Grade 3 and the Sorafenib treatment was discontinued. We then initiated treatment with a topical steroid, oral antihistamines and local management with ice packs around the lesions and observed the beginning of recovery seven days after the patient discontinued the drug therapy (Figure 2).

## Conclusions

Sorafenib (BAY 43-9006) is the first approved systemic therapy in the management of radioactive iodine (RAI)-resistant metastatic differentiated thyroid cancer. The use of Sorafenib, is however associated with a relatively high incidence of adverse events already described in dermatological clinical trials of this drug, but its use in medical practice, common in the patient with metastatic thyroid carcinoma has not yet been reported and this is one of the first post marketing reports.

Hand-foot syndrome (HFS) is a relatively frequent dermatologic toxic reaction to certain chemotherapies for cancer. Its pathogenesis is not well defined, it is usually mild, but can progress to a severe condition that limits function and affects quality of life. The most frequently used therapies are topical emollients, pain medications and reduction from the full dose or discontinuation of the Sorafenib therapy followed by resumption of treatment at 50% of the full dose often associated with good response rate.

Given the incidence of thyroid carcinoma, and the importance of Sorafenib as a new therapeutic option for patients with advanced radioactive iodine (RAI)-refractory desease, it is important that clinicians including endocrinologists, medical oncologists who manage these therapies are fully aware of the potential adverse effects. All patients on Sorafenib therapy should be educated to recognize the first symptoms, to manage initial prevention and consider dose reduction or discontinue the therapy to improve the patient’s quality of life by minimizing the impact of adverse events while obtaining the maximal benefit from this anti-neoplastic rescue therapy.

## Consent

Written informed consent was obtained from the patient for publication of this Case report and any accompanying images. A copy of the written consent is available for review by the Editor of this journal.

## Abbreviations

(HFS): Hand-foot syndrome; (VEGFR): Vascular endothelial growth factor receptor; (FDA): Food and Drug Administration; (PPE): Palmar-plantar Erythrodysesthesia; (PDGFR): Platelet-derived growth factor receptor; (RAI): Radioactive iodine.

## Competing interests

The authors declare that they have no political, personal, religious, ideological, academic, intellectual, commercial or any other competing interests.

## Authors’ contributions

CAVC carried out the: wrote the manuscript, data collection, reviewed and edited manuscript. EAWS carried out the: data collection reviewed and edited manuscript; DAGA carried out the reviewed and edited manuscript. All authors read and approved the final manuscript.

## Authors’ information

CAVC: Author; Internal Medicine Resident first year, Universidad Autónoma de Bucaramanga-UNAB.

EAWS: Author; Endocrinologist, Assistant Professor of Medicine, Universidad Autónoma de Bucaramanga-UNAB.

DAGA: Co-author; Medical Oncologist, Coordinator of the Department of Clinical Oncology, Cal ONCOLOGICOS, Bucaramanga, Colombia.

## Pre-publication history

The pre-publication history for this paper can be accessed here:

http://www.biomedcentral.com/1472-6823/14/26/prepub
